# Prevention of Dental Caries: A Review on the Improvements of Toothpaste Formulations from 1900 to 2023

**DOI:** 10.3390/dj12030064

**Published:** 2024-03-04

**Authors:** Patrick Unterbrink, Erik Schulze zur Wiesche, Frederic Meyer, Pascal Fandrich, Bennett T. Amaechi, Joachim Enax

**Affiliations:** 1 Research Department, Dr. August Wolff GmbH & Co. KG Arzneimittel, Sudbrackstr. 56, 33611 Bielefeld, Germany; patrick.unterbrink@drwolffgroup.com (P.U.); erik.schulzezurwiesche@drwolffgroup.com (E.S.z.W.); 2 Research Department, Dr. Kurt Wolff GmbH & Co. KG, Johanneswerkstr. 34–36, 33611 Bielefeld, Germany; frederic.meyer@drwolffgroup.com (F.M.); pascal.fandrich@drwolffgroup.com (P.F.); 3 Department of Comprehensive Dentistry, School of Dentistry, University of Texas Health San Antonio, 7703 Floyd Curl Drive, San Antonio, TX 78229-3900, USA; amaechi@uthscsa.edu

**Keywords:** toothpaste, dentifrice, active ingredient, caries, patent application, Espacenet, Mintel

## Abstract

Modern toothpastes are complex formulations with various ingredients. The aim of this study was to analyze the improvement of toothpaste formulations from 1900 to 2023 focusing on active ingredients with remineralizing, antibacterial, or plaque-removing effects, and to discuss their influence on caries prevention. For this, worldwide patent applications were searched using the international database Espacenet from the European Patent Office. Additionally, toothpaste products were searched using the Mintel product database from 1996 to 2023. The searched ingredients were (in alphabetical order): calcium carbonate, calcium phosphates, hydrated silica, sodium fluoride, sodium lauryl sulfate, triclosan, xylitol, and zinc salts as they are known from the scientific literature to be remineralizing or antibacterial/antiplaque agents. It was shown that the number of patent applications containing these ingredients significantly increased since the 1970s. As these ingredients have remineralizing, antibacterial, or plaque-removing effects, they all can contribute to caries prevention. In conclusion, and within the limitations of this approach, this study shows that toothpaste formulations have greatly improved over the past decades by using various active anticaries ingredients.

## 1. Introduction

The use of modern toothpastes is crucial to prevent both caries and gum diseases [1]. Since caries is caused by acids produced by cariogenic bacteria in the plaque on the tooth surface, there are several strategies for caries prevention [2]. Regarding toothpastes, this includes enhancing tooth remineralization, preventing tooth demineralization, the use of antibacterials, and an efficient removal of plaque using abrasives [2,3,4].

Among others, fluoride compounds such as sodium fluoride, stannous fluoride, amine fluoride, and sodium monofluorophosphate can be used in toothpastes for the prevention of dental caries [1,5]. Besides fluoridated toothpastes, fluoride-free toothpastes without or with proven anti-caries agents are also commercially available. Fluoride-free toothpastes can contain agents such as calcium phosphates or xylitol for protection against caries [6,7]. Calcium phosphates used in toothpastes for caries prevention are mainly hydroxyapatite (HAP), casein phosphopeptide–amorphous calcium phosphate (CPP-ACP), calcium sodium phosphosilicate (CSPS, or so called Bioglass), or β-tricalcium phosphate (β-TCP) [6].

Along with these agents, modern toothpastes may contain a variety of other active ingredients that can contribute to caries prevention, such as tooth cleaning agents (i.e., abrasives) that, in combination with the toothbrush, lead to plaque (and stain) removal [1,8], and agents with antibacterial properties to combat cariogenic bacteria [1,4,9].

Common abrasives in toothpastes are hydrated silica and calcium carbonate [1,8], and frequently used antibacterial agents are zinc salts (e.g., zinc citrate or zinc chloride) and stannous salts (e.g., stannous fluoride or stannous chloride) [1,9]. In addition to these active ingredients, toothpastes also contain various classes of ‘adjunct’ ingredients, including viscosity and rheology modifiers, humectants, flavors, sweeteners, coloring agents, preservatives, and water [1].

Besides toothpastes used for caries protection, “all-in-one” toothpastes and toothpastes for further indications, such as to relief dentin hypersensitivity, for tooth whitening, and for improving gingival health, are also available on the market [1,10,11].

Toothpaste formulations have greatly improved within the last decades [12,13,14,15]. At the beginning of the 19th century, homemade tooth powders were commonly used by many people to clean their teeth [15]. Those tooth powders contained mainly basic ingredients such as precipitated chalk and soap. To mask the bad taste (due to the soap), saccharin and even sucrose were added [15] (Table 1).

The formulations of those tooth powders were far simpler than the advanced toothpaste formulations sold today.

The first toothpaste formulation with fluoride described in the patent literature is shown in Table 2. It is worth noting that this toothpaste formulation did not include many common ingredients found in toothpastes today.

Various ingredients are used in toothpastes [1]. Nowadays, toothpastes can also contain ‘new’ classes of ingredients such as minerals (e.g., calcium phosphates), enzymes/proteins (e.g., lactoferrin, amyloglucosidase, glucose oxidase, and lactoperoxidase), or natural extracts (e.g., bisabolol) [6,17]. Moreover, besides sodium lauryl sulfate, various other surfactants are used in modern toothpastes such as cocamidopropyl betaine or certain types of sarcosinates [1]. Taken together, this shows that modern toothpaste formulations are highly complex and can be tailored to meet the specific needs of the respective target group.

There are a few publications that have described the development of toothpastes [12,13,14,15]. Fischman described the change in composition of oral care products including toothpastes, mouthwashes, and toothbrushes over a long period of time (starting 4000 B.C.E.) [12]. Interestingly, the first tooth cleaning formulations are mentioned as early as 4000–1500 B.C.E. [12] Another review on the long history of toothpastes was published by Lippert [13]. It described that the very first tooth cleaning formulations “…contained powdered ashes from oxen hooves, myrrh, eggshells, and pumice…” [13]. These early approaches can be seen as precursors to the development of modern tooth cleaning agents [8]. The review written by Lippert also highlights some milestones of toothpaste development from 1914 onwards [13]. Another review on toothpaste improvement was published by Madhuri and Buggapati [14]. It also gives an overview of the development of toothpastes over a long period of time [14]. In addition to these review articles, a book on the general improvements of toothpastes and toothbrushes in the USA from 1900 to 2008 was published [15]. It describes some examples of the commercial development of toothpastes and toothbrushes, including advertisements during this period [15].

Taken together, the historic development of toothpastes over a long period of time (i.e., starting from B.C.E.) is well-described in the literature. However, to date, a detailed analysis of the improvement of toothpaste formulations with a special focus on the period from 1900 to 2023 is missing. This period is of particular interest because a major decline in caries prevalence has been observed [3,18]. However, the prevalence of caries remains high among both children and adults.

To address this gap, this study aims to analyze improvements in toothpaste formulations during this period.

To carry out an innovative investigation, global patent applications were searched from 1900 to 2023. Additionally, commercial toothpaste products were examined from 1996 to 2023, as earlier records were not available in the utilized database. This article focuses on selected active ingredients commonly used in modern toothpastes for their remineralizing, antibacterial, or plaque-removing effects such as calcium carbonate, calcium phosphates, hydrated silica, sodium fluoride, sodium lauryl sulfate, triclosan, xylitol, and zinc salts. In addition, this study will analyze the potential contribution of these ingredients to the decrease in caries prevalence over the last decades.

## 2. Materials and Methods

### 2.1. Concept and Active Ingredients

The basis for the present study is built upon the concept of so-called “innovation studies” with a focus on the output type, namely patent applications and products, related to the development of interest. These are measurable considering the number per year either published or introduced. As described above, these numbers will be used to evaluate the improvement of toothpastes over the mentioned time period.

Active ingredients commonly found in modern toothpastes with remineralizing, antibacterial, or plaque-removing effects were searched [1], i.e., calcium carbonate, calcium phosphates, hydrated silica, sodium fluoride, sodium lauryl sulfate, triclosan, xylitol, and zinc salts.

### 2.2. Selection of Patent Database

There are various patent databases that have the potential to be used for this study. Therefore, five common patent databases were compared, considering the total number of patent applications, regarding the search string “toothpaste” between 1900 and 2023, as well as the earliest available patent application within this time frame (Table 3). All searches were conducted on 8 December 2023.

Despite the fact that the highest number of results were found using Google Patents and DEPATISnet, Espacenet was considered more suitable for this study as the main patent authorities are covered and this database includes patents from 1854 on (Table 3). It is important to note that Google Patents is not only displaying true results (i.e., the searched patent applications), but also contains related or cited patent applications, explaining the significant difference in the number of patents found. DEPATISnet also offers great authority coverage as well as a high number of patent applications (higher than Espacenet), but the record starts in 1933, which is the reason why it was found to be not suitable for this study.

### 2.3. Worldwide Patent Search Using Espacenet (1900 to 2023)

For the patent search, the online database Espacenet from the European Patent Office (version 1.45.0, https://worldwide.espacenet.com/) was used. The search was conducted on 2 January 2024.

All searches mentioned here were conducted using the “nftxt” term of Espacenet which includes all text fields and name fields.

The search strategy is summarized in Table 4. In a first step, the total number of patent applications containing the search terms “toothpaste” or “dentifrice” was collected. Second, the synonyms and the corresponding search strings for each ingredient were defined (Table 5) and added via Boolean operators to the term. Third, the timeframes were restricted to certain decades starting with 1900 to 1909 and ending with 2010 to 2019, reflected by the operator “pd within”. The period 2020–2023 was also searched, but is not mentioned at this point, as it does not represent a whole decade.

It was searched for the whole terms, decade-wise, and the numbers of patent applications found were recorded in Tables S1–S3 (Supplementary Materials).

In addition to sodium lauryl sulfate, certain other surfactants were searched using the search terms “cocamidopropyl betaine”, “sodium myristoyl sarcosinate”, “sodium methyl cocoyl taurate”, and “sodium cocoyl glycinate”.

### 2.4. Mintel Search

To broaden the scope of this study, an additional search using the Mintel product database was conducted (https://www.gnpd.com/sinatra/gnpd/search&search_id=oTfwareEgj/ accessed on 2 January 2024). The search terms for the Mintel search were developed according to the terms used for the Espacenet search with one difference (Table 6), as the synonyms were chosen from the ingredients list, offered by Mintel. The Mintel search was limited to toothpaste products which have been commercially available since 1996 as earlier products were not available in this database. This search was also conducted on 2 January 2024.

### 2.5. Mintel Search Germany Compared with Caries Prevalence in Germany within 1997–2014

A similar search as described in Section 2.4, using the Mintel product database and the same ingredients, was conducted, but this time restricted to the German market explicitly, as the aim was to compare these results with representative data of the caries prevalence of 12-year-olds in Germany between 1997 and 2014 [18]. This search was also conducted on 2 January 2024.

### 2.6. Worldwide Mintel Search—Hydroxyapatite (1996 to 2023)

Another search, using the same procedure as in 2.4, for toothpaste products containing hydroxyapatite, was conducted. The synonyms used were: “calcium/magnesium/zinc hydroxyapatite”, “calcium hydroxyapatite”, “zinc hydroxyapatite”, and “hydroxyapatite”. This search was also conducted on 2 January 2024.

### 2.7. Presentation of Data

The main results are presented in the figures and tables. Please note that different colors in the graphics indicate different ingredients as shown in the legend. The dotted lines represent no causal connection and only facilitate visualization of the results. In addition, all search results are presented with tables in the Supplementary Tables S1–S3.

## 3. Results

### 3.1. Number of Worldwide Patent Applications per Decade (1900 to 2019)

**Figure 1:**
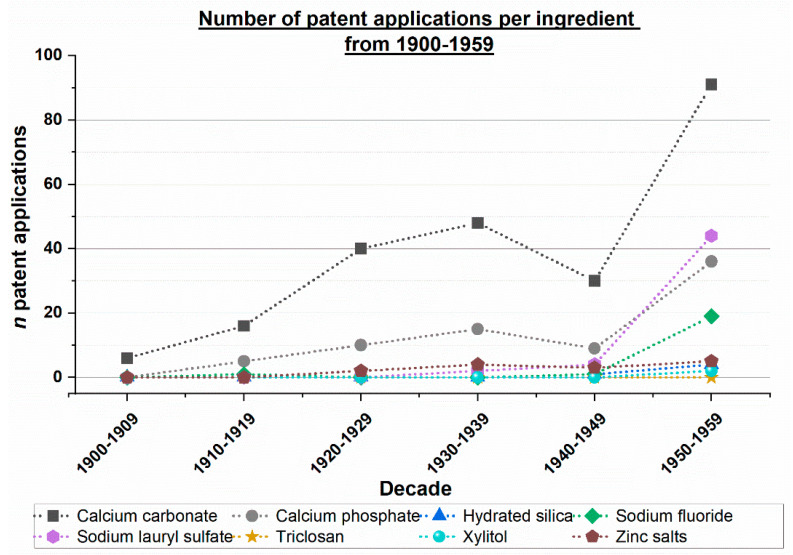
Number of worldwide patent applications per decade from 1900 to 1959 per ingredient.

**Figure 2:**
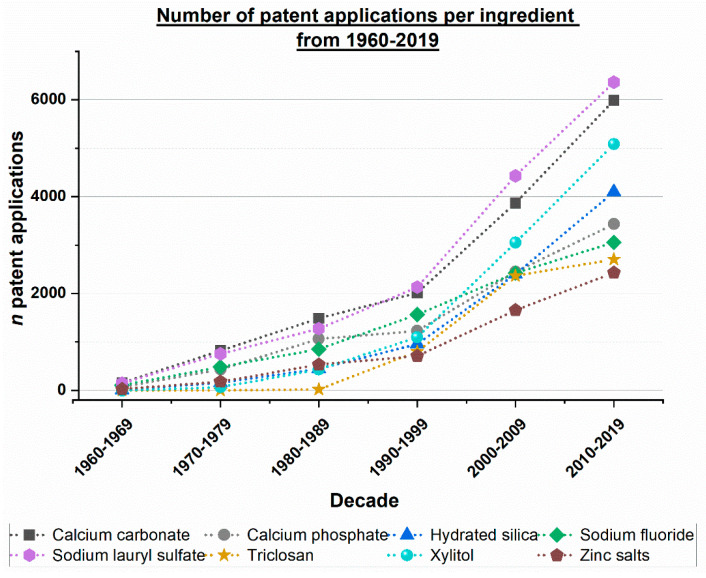
Number of worldwide patent applications per decade from 1960 to 2019 per ingredient.

The Espacenet search for patent applications was conducted as described above. A huge difference in the total number of patent applications over the course of the years was observed, leading to an illustration of the results, split into two separate figures, i.e., one covering the time period from 1900 to 1959 and a second covering the time period from 1960 to 2019 (Figure 1 and Figure 2).

In the period from 1900 to 1959 the maximum number of patent applications includes calcium carbonate, calcium phosphate, and sodium lauryl sulfate as ingredients, with the highest number of patent applications between 1950 and 1959 of 91 applications containing calcium carbonate (Figure 1).

Taking the total number of patent applications (1900 to 2019) into account, it is possible to observe a major difference between the periods 1960 to 1969 and 1970 to 1979: the number of applications is eight times greater than that of the previous decade. In addition, this point in time seems to mark the start of rising patent application numbers in general. There is a difference between the number of patent applications before 1969 and thereafter, resulting in two distinct periods (I and II). These results are shown in Table S1.

### 3.2. Analysis of Toothpastes Products (1996 to 2023)

**Figure 3:**
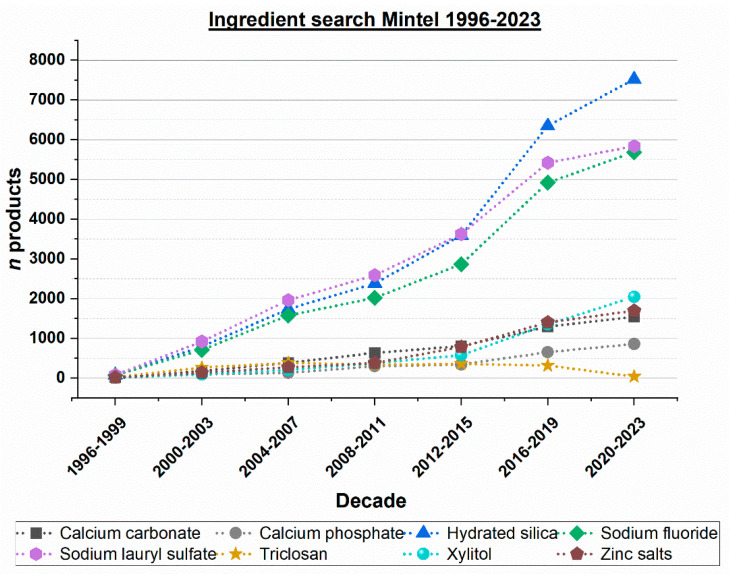
Worldwide ingredient search for the period of 1996 to 2023, toothpastes on the market containing certain ingredients.

Furthermore, additional data from the Mintel product database, considering the period of 1996 to 2023 and the ingredients mentioned, were collected. These results show a clear separation within the group of investigated ingredients (Figure 3).

There are a lot more toothpaste products containing hydrated silica, sodium lauryl sulfate, or sodium fluoride compared to the other ingredients. Nevertheless, for almost all ingredients, a clear trend of increasing product numbers can be observed. The numbers of toothpaste products containing triclosan, however, have decreased in recent years (Figure 3).

### 3.3. Analysis of Toothpaste Products Containing Hydroxyapatite (1996 to 2023)

**Figure 4:**
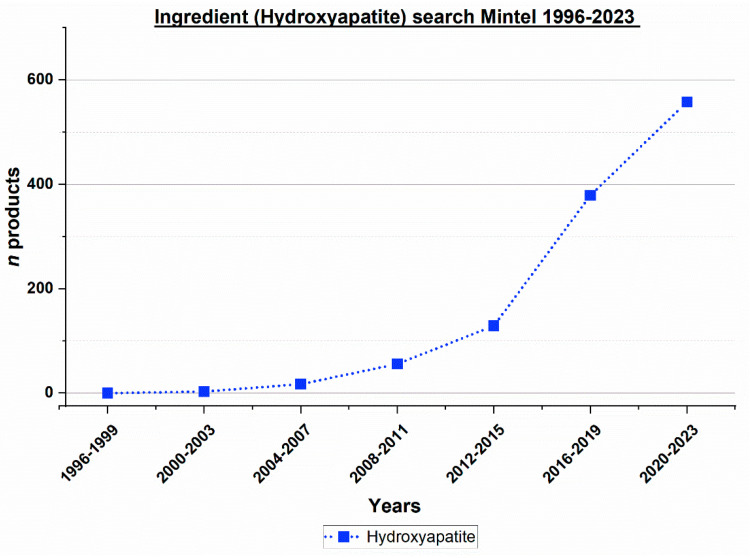
Worldwide ingredient search for the period of 1996 to 2023, toothpastes on the market containing hydroxyapatite.

The class of calcium phosphates contains different substances [19]. Out of different calcium phosphates, hydroxyapatite has been most extensively studied in clinical studies in the field of oral care [6,20]. Therefore, the development of the number of toothpaste products containing hydroxyapatite was analyzed (Figure 4). Since 2008, there has been a significant trend towards an increase in the number of toothpaste products containing hydroxyapatite (Figure 4).

### 3.4. Analysis of Toothpaste Products within the German Market and Comparison with Caries Prevalence (1996 to 2014)

**Figure 5:**
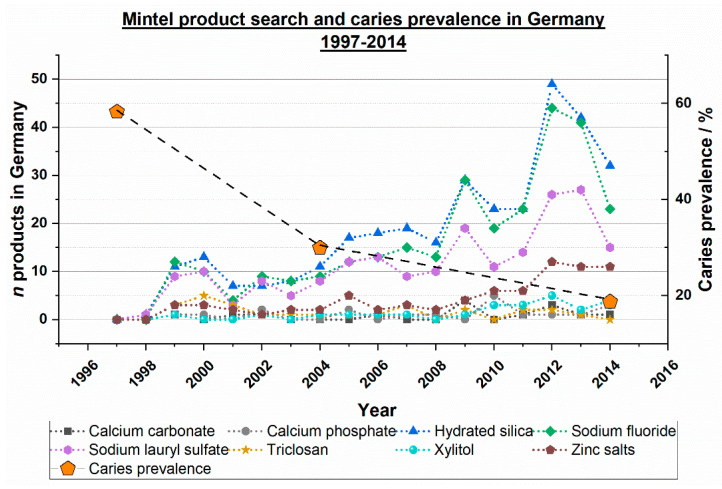
Ingredient search for the period 1996 to 2023, toothpastes on the German market containing certain ingredients. Orange pentagons showing the results of each German oral health study within this period (caries prevalence of 12-year-olds) [18].

Considering the results of the product search on the German market, there are also increasing numbers of the products as seen in the worldwide development, but there is not such a clear separation of the numbers of toothpaste products between the mentioned ingredients. There is a clear trend of a reduced caries prevalence in 12-year-olds in Germany (Figure 5).

Besides sodium lauryl sulfate, other surfactants frequently used in modern toothpastes were searched and a trend towards a higher number of patent applications containing these agents could be observed (Table 7).

## 4. Discussion

### 4.1. Improvement of Toothpaste Formulations from 1900 to 2023—General Considerations

Analyzing patent applications can be used to monitor technical developments and innovations [21]. This kind of analysis has been used for different aims “such as monitoring technology trends, analyzing technology innovation patterns or developing technology strategies” [21].

While some papers cover the extensive history of toothpaste development [12,13,14,15], this study is the first of its kind to analyze toothpaste ingredients by searching worldwide patent applications (from 1900 to 2023) and commercial toothpaste products (from 1996 to 2023).

The results show that two main periods can be identified, i.e., period I from the 1900s to the 1960s and period II from the 1970s to present day (Figure 1 and Figure 2). Agents that are commonly used in toothpaste formulations today have not all been described in patent applications before the 1970s. Table 1 and Table 2 provide an example of an ‘early’ tooth powder and toothpaste formulation from the early 1900s. The formulation presented in these tables did not contain ingredients such as abrasives or antibacterial agents used in toothpastes nowadays. Beginning in the 1950s, there was a notable increase in the number of patent applications containing ingredients with remineralizing, antibacterial, and plaque-removing effects (Figure 1). Starting around the 1970s, toothpaste formulations have undergone significant improvements (Figure 2).

Caries is a multifactorial disease; i.e., it involves the interplay of teeth, cariogenic bacteria in plaque, and dietary sugars [2,3]. The searched active ingredients (i.e., calcium carbonate, calcium phosphates, hydrated silica, sodium fluoride, sodium lauryl sulfate, triclosan, xylitol, and zinc salts) have remineralizing effects, antibacterial effects, or plaque-removing properties. Thus, they can all contribute to caries prevention (Table 8) [2].

The modes of action of the searched active ingredients in caries prevention are briefly described below (note that the ingredients are alphabetically ordered).

(A) Calcium carbonate

Calcium carbonate is a relatively soft mineral with abrasive effects, which allows gentle plaque removal [8]. Based on its chemistry, it can also buffer acids and release calcium ions under acidic conditions. In toothpastes, calcium carbonate is used in the modification of calcite [8].

(B) Calcium phosphate

Different calcium phosphates are used in toothpastes for caries prevention [6]. This includes hydroxyapatite (HAP), casein phosphopeptide–amorphous calcium phosphate (CPP-ACP), calcium sodium phosphosilicate (CSPS, or so called Bioglass), β-tricalcium phosphate (β-TCP), and calcium glycerophosphate (CaGP) [6]. Calcium phosphates have remineralizing effects on enamel and dentin [6,24], and they are also suitable for remineralization of teeth affected by molar incisor hypomineralization (MIH) [25]. Moreover, some of these calcium phosphates also have plaque-removing properties [8]. Calcium phosphates have an excellent biocompatibility. While not being biocide, calcium phosphates can reduce the bacterial colonization to tooth surfaces [26].

(C) Hydrated silica

Besides calcium carbonate, hydrated silica is one of the most frequently used abrasives [1]. It is important to note that hydrated silica represents a class of compounds that can vary through various factors such as water content, particle form, and particle size distribution [8]. Silica abrasives are highly efficient in plaque removal while being gentle to teeth and gingiva [27]. Silica abrasives have a special microscopic structure that has been optimized for tooth cleaning [8]. For further details on the improvements of abrasives in general, see the text below.

(D) Sodium fluoride

Sodium fluoride is one of the most frequently used fluoride compounds in fluoridated toothpastes [1]. The addition of fluoride to toothpaste reduces the caries risk [5]. Fluorides have both remineralizing and antibacterial effects [1,22]. Certain counterions of various fluoride compounds can increase the antibacterial effect of fluoride, as seen, e.g., in stannous fluoride and amine fluorides.

(E) Sodium lauryl sulfate

Sodium lauryl sulfate is an anionic surfactant and the most widely used surfactant in toothpastes today [1]. Surfactants are used to disperse the active ingredients in the oral cavity and to support the cleaning efficacy of toothpastes (e.g., by removing hydrophobic compounds from the tooth surface). Additionally, sodium lauryl sulfate has antibacterial properties [9].

(F) Triclosan

Triclosan has antibacterial properties, including activity against *Streptococcus mutans* [9,28]. However, in recent years, there has been controversy about its safety [29]. There are suspected health issues associated with long-term use of triclosan-containing products as published by the United States Food and Drug Administration [30].

(G) Xylitol

Xylitol is both a caries-preventing agent [7], and a non-cariogenic sweetener in toothpaste formulations [3]. Xylitol has various modes of actions in the oral cavity, and one is that it can lead to a reduction of levels of mutans streptococci [23].

(H) Zinc salts

Zinc salts, such as zinc chloride and zinc citrate, are commonly used in toothpastes [1]. They have been shown to have antibacterial properties (e.g., against *S. mutans*) [9,31,32]. In addition, zinc salts are used to prevent calculus formation and halitosis [1]. In contrast to stannous salts, zinc salts do not stain the tooth surface [32].

General considerations regarding toothpaste abrasives

The technology of toothpaste abrasives has continuously improved. For example, a comprehensive in vitro study analyzed cleaning efficacy (PCR; pellicle cleaning ratio) and dentin abrasion (RDA; radioactive dentin abrasion) of 41 toothpastes which were available on the European market in 1995 [27]. The author compared the results to data from 1988 and found that there was an overall trend towards toothpastes with lower dentin abrasion values without a significant loss of cleaning power; i.e., toothpastes became more efficient from 1988 to 1995. This trend can be explained by the progress in the development of toothpaste abrasives, mainly hydrated silica [8,27]. For example, hydrated silica (PCR/RDA ratio 2.6) has a higher cleaning performance to dentin abrasion ratio than dicalcium phosphate dihydrate (PCR/RDA ratio 1.6) [27]. Both the number of patent applications containing hydrated silica as well as the number of toothpaste products with hydrated silica have significantly increased over the last decades (Figure 2, Figure 3 and Figure 5).

Moreover, another reason for the increase in the number of patent applications and toothpaste products is that hydrated silica can be formulated with fluoride [8] (the trends for sodium fluoride and hydrated silica are almost identical as shown in Figure 3). In contrast, fluoride should not be formulated with calcium-containing abrasives such as calcium carbonate or calcium phosphates due to the possible formation of insoluble calcium fluoride [8,33].

Complexity of toothpaste formulations

The complexity of toothpaste formulations has greatly increased over the last decades. This has become visible in the increase of both the number of patent applications and toothpaste products containing remineralizing, antibacterial, or plaque-removing effects over the last decades (Figure 1, Figure 2, Figure 3 and Figure 4). Additionally, numerous other ingredients have gained increasing popularity in toothpaste formulations. For example, various types of surfactants have been described recently (Table 7). They can be used alone or in combinations, in addition to sodium lauryl sulfate or as substituents.

### 4.2. Improvement of Toothpaste Formulations from 1900 to 2023—Impact on Caries Decline

In a review article published in 1996 entitled “The caries decline: a review of reviews”, Petersson and Bratthall concluded that “… various authors believe that the use of fluoride in various forms has contributed most significantly to the decline in dental caries prevalence. A number of other factors must, however, also be taken into consideration …” [34]. However, this review does not contain deeper analyses but only presents expert opinions and does not include any calculations on the impact of individual factors on the decline of caries [34]. To the best of the authors’ knowledge, no published study has quantitatively analyzed individual factors such as fluorides, calcium phosphates, antibacterial agents, plaque-removing toothpaste agents (abrasives), toothbrushes, fissure sealants, diet, sugar taxes, oral hygiene programs, and regular dental checkups etc. on the decline of dental caries in different countries.

Based on the analysis of patent applications on toothpastes, the present study demonstrated that toothpaste formulations have significantly improved since the 1970s. Therefore, to accurately estimate the influence of toothpastes on the global decline in caries, it is not sufficient to rely solely on sales and the global availability of fluoride toothpastes as previously performed by experts [34]. A thorough analysis of the contribution of each toothpaste ingredient on caries decline should be conducted in future.

Figure 5 illustrates the relationship between the number of toothpaste products in Germany containing ingredients that can impact caries and the prevalence of caries in Germany (Germany was chosen as an example of a highly developed industrialized country). The figure shows that not only fluorides, but also other agents, mainly hydrated silica and sodium lauryl sulfate, may have contributed to the global decline in caries within the last decade (see also Table 8).

**Figure 6:**
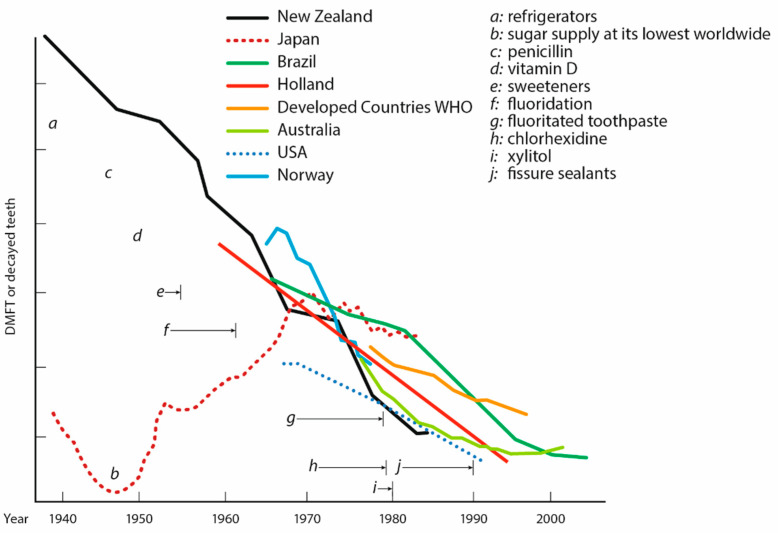
Overview of the global decline in caries and factors that may have contributed to this decline (DMFT: Decayed, Missing, Filled Teeth). The horizontal arrows represent the periods in which these factors were introduced. The original graphic can be found in reference [3]. In our study, it was shown that the number of patent applications on toothpastes containing ingredients with remineralizing, antibacterial, or plaque-removing effects significantly increased since the 1970s (Figure 2). It is worth mentioning that the very first patent applications describing ingredients such as hydrated silica, sodium lauryl sulfate, or zinc salts were mentioned already in the period around the 1940s to the 1950s (Supplementary Materials Table S1).

The results of the present study support the influence of fluoride on caries decline [34] (Figure 6). However, it should be noted that fluorides were already introduced in commercial toothpastes in the 1950s [13]. Therefore, fluorides may not be the only factor contributing to the global decline in caries, particularly in recent decades. It is noteworthy that the increase in toothpaste products containing sodium fluoride has been paralleled by a similar increase in products containing hydrated silica and sodium lauryl sulfate, both of which can also contribute to the caries-preventing effect of toothpaste, see above (Figure 3 and Table 8).

It is worth mentioning that the use of toothpastes has not only had an influence on the global caries decline. Figure 6 also illustrates some of the other proposed factors which may have contributed to a reduced global caries prevalence [3].

The discussion of all factors that have contributed to the global decline in caries is beyond the scope of this study because this study focuses on toothpastes only. However, it is important to note that future studies should also analyze factors such as fissure sealants, diet, sugar taxes, regular dental visits, professional tooth cleaning, and the use of electric toothbrushes.

The present study provides additional information on the development of toothpastes by analyzing patent applications and toothpaste products. It is likely that various ingredients such as antibacterial agents, calcium phosphates, and abrasives have contributed to the global decline in caries. This is due to the etiology of caries, which requires the presence of bacteria and plaque for caries to develop [35]. Calcium phosphates provide calcium and phosphate ions for remineralization [6], antibacterial agents combat cariogenic bacteria [4,36], and abrasives and surfactants remove plaque [8]. Hydroxyapatite, for example, has been incorporated in an increasing number of toothpaste products (Figure 4). Clinical studies have shown that fluoride-free hydroxyapatite-based toothpastes prevent caries [6,37].

### 4.3. Limitations

#### 4.3.1. Search for Patent Applications

Due to the high number of patent applications in the field of toothpastes, only a full-text search was performed, which means that the role of the ingredients in each patent application could not be specified (i.e., in some cases, the ingredients being searched for may not be listed as part of the toothpaste formulation). In some cases, the active ingredients are described in combination with other ingredients. Although common synonyms for some of the searched ingredients were used, it cannot be fully excluded that the search was Incomplete due to various chemical names and trade names of certain ingredients. Furthermore, only a selection of toothpaste ingredients was analyzed. While this review focused on some of the main active ingredients used in toothpastes, future analyses of patent applications could broaden the scope by including additional ingredients (e.g., further fluoride compounds or calcium compounds). A detailed analysis of the patent applications was not performed, such as analyzing the details of the applicants and inventors or providing an overview of the countries in which the patents were granted. It is possible that a patent application may contain several of the searched ingredients. Furthermore, in some patent applications, “text blocks” of previous patent applications may have been used; i.e., these patent applications would not describe “new” data. Finally, the mention of an active ingredient in a patent application does not necessarily mean that this active ingredient will be used in a commercial product.

#### 4.3.2. Search for Toothpaste Products

A limitation of the Mintel search was that only toothpaste products from 1996 were available in this database. Furthermore, although this database contains toothpastes from all over the world, it does not cover all toothpastes which have been globally introduced.

## 5. Conclusions

Toothpastes are complex formulations with various ingredients. Active ingredients commonly used for their remineralizing, antibacterial, and plaque-removing effects were analyzed. The analysis of worldwide patent applications in the toothpaste field reveals two main periods: period I from the 1900s to the 1960s, and period II beginning in the 1970s. Since the 1970s, there has been a significant increase in the number of patent applications featuring the active ingredients under investigation, including calcium carbonate, calcium phosphates, hydrated silica, sodium fluoride, sodium lauryl sulfate, triclosan, xylitol, and zinc salts. In contrast, tooth powders and toothpastes commonly used in the early 1900s lacked these active agents. Beginning in the 1950s, there was a notable increase in the number of patent applications containing ingredients with remineralizing, antibacterial, and plaque-removing effects.

Commercial toothpaste products were analyzed from 1996 to 2023 (as earlier data were not available in the database used) and the general trend found in the patent applications could be confirmed. In particular, the number of toothpaste products containing hydrated silica, sodium lauryl sulfate, and sodium fluoride has increased globally. Globally, the increase in toothpaste products containing calcium carbonate, calcium phosphates, xylitol, and zinc salts is less pronounced than that of products with hydrated silica, sodium lauryl sulfate, and sodium fluoride. However, interest in these ingredients has also continued to increase. Caries is a multifactorial disease, the development of which involves the interplay of teeth, cariogenic bacteria in plaque, and dietary sugars. Since the searched toothpaste ingredients have remineralizing, antibacterial, or plaque-removing effects, they all can contribute to caries prevention.

In conclusion, and within the limitations of this approach, this study shows that toothpaste formulations have greatly improved over the past decades by using various active anti-caries ingredients.

## Figures and Tables

**Table 1:** Example of a tooth cleaning powder used in the USA in the early 1900s (taken from ref. [15]). The tooth cleaning properties can be assigned to precipitated chalk (i.e., calcium carbonate) and soap. Note that even cariogenic (sucrose) sugar was added to the formulation.

Ingredient (in Descending Order by Weight Percentage in the Tooth Powder Formulation)	Percentage/wt%
Precipitated chalk	79.0
Florentine orris	9.9
White castile soap	4.9
Sugar	4.9
Oil of wintergreen	1.2

**Table 2:** Example of a toothpaste formulation described in the first patent application mentioning fluoride in toothpastes 1914 (modified from: GB191403034A; applicant and inventor: Cecil Rudolph Lidgey, UK) [16].

Ingredient (in Descending Order by Weight Percentage in the Toothpaste Formulation)	Percentage/wt%
Calcium fluoride	40.05
Calcium phosphate	26.70
Glycerol	26.70
Potassium chlorate	2.67
Sodium fluoride	1.34
Ammonium fluoride	1.34
Menthol	0.40
Oil of cinnamon	0.40
Essence of peppermint	0.40

**Table 3:** Comparison of different patent databases for the search string “toothpaste”. Espacenet, Lens, PatentScope, GooglePatents, and DEPATISnet were compared in terms of total number of patent applications within the period from 1900 to 2023 and the earliest patent application considering the search for toothpaste.

Database	Espacenet	Lens	PatentScope	Google Patents	DEPATISnet
Number of results from 1900 to 2023	78,377	72,970	68,545	239,006	127,289
Year of earliest patent application	1854	1913	1901	1923	1933

**Table 4:** Details of the search strategy.

Total Number of Patent Applications Including Toothpaste or Dentifrice	nftxt all “toothpaste” OR nftxt all “dentifrice”
Adding ingredient search strings	AND nftxt = “Example ingredient” OR nftxt = “Another example ingredient”
Search for each decade (1900 to 2019)	pd within “1900,1909” (etc.)
Collect the number of	1st- total number/2nd- total number + ingredient

**Table 5:** Development of ingredient synonyms and search strings: left column shows the ingredients with their synonyms, right column shows the “translation” of synonyms into search strings used for the search for patent applications.

Ingredients (Alphabetically Ordered)	Synonyms Used in Search
Calcium carbonate	“calcium carbonate”, “calcite”, “chalk”
Calcium phosphate	“calcium phosphate”
Hydrated silica	“hydrated silica”, “precipitated silica”, “silica abrasive”, “silica dental abrasive”, “silicon dioxide”, “zeodent”, “sylodent”
Sodium fluoride	“sodium fluoride“
Sodium lauryl sulfate	“sodium lauryl sul*”, “sodium dodecyl sul*”, “SLS”, “SDS”, “sodium laurilsul*”
Triclosan	“triclosan”, “irgasan”
Xylitol	“xylitol”, “xylit”, “E967”
Zinc salts	“zinc acetate”, “zinc chloride”, “zinc glu-conate”, “zinc sul*”, “zinc citrate”, “zinc lactate”

**Table 6:** Development of ingredient synonyms and search strings for Mintel search.

Ingredient (Alphabetically Ordered)	Synonyms Used in Mintel Search
Calcium carbonate	“calcium carbonate”, “calcite”, “CI 77220”
Calcium phosphate	“calcium phosphate”, “dicalcium phosphate”, “dicalcium phosphate dihydrate”, “hydroxyapatite”, “tricalcium phosphate”
Hydrated silica	“hydrated silica”, “hydrated silica (Zeodent 113)”
Sodium fluoride	“sodium fluoride”
SLS	“SLS”, “SDS”, “Sodium lauryl sulfate (SLS)”
Triclosan	“triclosan”, “triclosan (irgasan)”
Xylitol	“xylitol”
Zinc salts	“zinc acetate”, “zinc chloride”, “zinc gluconate”, “zinc sulfate”, “zinc citrate”, “zinc lactate”

**Table 7:** Overview of the number of patent applications containing certain surfactants (from 1900 to 200 and 2001 to 2023).

Surfactant/Period	Cocamidopropyl Betaine	Sodium Myristoyl Sarcosinate	Sodium Methyl Cocoyl Taurate	Sodium Cocoyl Glycinate	Sodium Lauryl Sulfate
1900 to 2000	90	8	37	0	3632
2001 to 2023	966	103	388	155	9267

**Table 8:** Overview of modes of action of the searched toothpaste ingredients in caries protection.

	Modes of Action in Caries Prevention			
Ingredients (Alphabetically Ordered)	Remineralization Effects	Antibacterial Effects	Plaque-Removing Effects	Literature
Calcium carbonate	---	---	Yes	[8]
Calcium phosphates *	Yes	---	Yes	[6,8]
Hydrated silica	---	---	Yes	[8]
Sodium fluoride	Yes	Yes	---	[1,22]
Sodium lauryl sulfate	---	Yes	Yes	[1,9]
Triclosan	---	Yes	---	[9]
Xylitol	---	Yes	---	[23]
Zinc salts	---	Yes	---	[9]

* Note that the class calcium phosphate contains different compounds [19].

## Data Availability

Not applicable.

## Supplementary Materials

The following supporting information can be downloaded at: https://www.mdpi.com/article/10.3390/dj12030064/s1, Table S1: Search results for patent applications in the field of toothpastes, Table S2: Search results for toothpaste products, Table S3: Search results for toothpaste products in Germany and the development of caries prevalence in 12-year-olds in Germany.
