# Supplementary material for: Prevention of Dental Caries: A Review on the Improvements of Toothpaste Formulations from 1900 to 2023
Source: Dent J (Basel). 2024 Mar 4;12(3):64. doi: 10.3390/dj12030064 (PMC10969581; doi:10.3390/dj12030064)
Supplement: Supplementary file 1 [file dentistry-12-00064-s001.zip › dentistry-2865769-supplementary.pdf]

Table S1. Search results for patent applications in the field of toothpastes (Espacenet, January 2, 2024).

Ingredient: Decade	Calcium Carbonate	Calcium Phosphate	Hydrated Silica	Sodium Fluoride	Sodium Lauryl Sulfate	Triclosan	Xylitol	Zinc salts
1900–1909	6	0	0	0	0	0	0	0
1910–1919	16	5	0	1	0	0	0	0
1920–1929	40	10	0	0	0	0	0	2
1930–1939	48	15	0	0	2	0	0	4
1940–1949	30	9	1	1	4	0	0	3
1950–1959	91	36	4	19	44	0	2	5
1960–1969	160	68	10	109	149	1	1	29
1970–1979	820	435	160	486	753	1	67	179
1980–1989	1487	1063	445	854	1275	22	432	534
1990–1999	2013	1227	957	1564	2132	802	1098	711
2000–2009	3868	2449	2399	2423	4429	2367	3050	1658
2010–2019	5984	3434	4099	3056	6360	2707	5084	2431
2020–2023	3004	1873	2200	1468	2814	1173	2744	1294

Table S2. Search results for toothpaste products (Mintel, January 2, 2024).

Ingredient: Years	Calcium Carbonate	Calcium Phosphate	Hydrated Silica	Sodium Fluoride	Sodium Lauryl Sulfate	Triclosan	Xylitol	Zinc Salts	Hydroxyapatite
1996–1999	17	6	74	72	75	24	13	16	0
2000–2003	177	94	791	709	918	264	92	136	3
2004–2007	384	131	1735	1576	1960	384	201	270	17
2008–2011	634	296	2381	2018	2586	336	375	378	56
2012–2015	802	343	3589	2865	3622	360	576	782	129
2016–2019	1294	652	6346	4921	5419	314	1347	1399	379
2020–2023	1544	857	7521	5658	5838	38	2044	1696	558

Table S3. Search results for toothpaste products in Germany and the development of caries prevalence in 12-year-olds in Germany [18] (Mintel, January 2, 2024).

Ingredient: Decade	Calcium Carbonate	Calcium Phosphate	Hydrated Silica	Sodium Fluoride	Sodium Lauryl Sulfate	Triclosan	Xylitol	Zinc Salts	Caries Prevalence/% (Germany)
1997	0	0	0	0	0	0	0	0	58.2
1998	0	0	1	0	1	0	0	0	
1999	1	1	11	12	9	3	1	3	
2000	0	1	13	10	10	5	0	3	
2001	1	0	7	4	3	3	0	2	
2002	1	2	7	9	8	1	1	1	
2003	0	0	8	8	5	1	0	2	
2004	0	0	11	9	8	1	1	2	29.9
2005	0	2	17	12	12	1	1	5	
2006	1	0	18	13	13	1	1	2	
2007	0	1	19	15	9	3	1	3	
2008	0	1	16	13	10	0	0	2	
2009	4	0	29	29	19	2	1	4	
2010	0	5	23	19	11	0	3	6	
2011	1	1	23	23	14	2	3	6	
2012	3	1	49	44	26	2	5	12	
2013	1	1	42	41	27	1	2	11	
2014	1	3	32	23	15	0	4	11	18.7
